# Low contrast visual acuity in healthy adults: Impact on non-pathologic myopia

**DOI:** 10.1371/journal.pone.0357713

**Published:** 2026-09-21

**Authors:** Toshio Narimatsu, Noriko Onozato, Haruna Togawa, Shigeto Shimmura, Yoko Ozawa

**Affiliations:** 1 Department of Clinical Regenerative Medicine, Fujita Health University School of Medicine, Ota, Tokyo, Japan; 2 Fujita Medical Innovation Center Tokyo, Ota, Tokyo, Japan; 3 Eye Center, Fujita Health University Haneda Clinic, Ota, Tokyo, Japan; 4 Department of Ophthalmology, Keio University School of Medicine, Shinjuku, Tokyo, Japan; Yokohama City University, JAPAN

## Abstract

Increase in myopia is a social issue worldwide. However, whether non-pathologic myopia with normal best-corrected visual acuity (BCVA) exhibits differences in visual ability is not well-documented. Data of 43 healthy eyes in 43 participants (52.8 ± 6.07 years) including 13 (30.2%) males who underwent eye medical checkups at Fujita Medical Innovation Center, Tokyo were retrospectively analyzed. Eyes with general BCVA 0 in logMAR or better and without histories of ocular diseases and surgeries were included. Mean low-contrast visual acuity (LCVA) measured using grey-scale optotypes with best correction was 0.17 ± 0.13 (range, −0.079–0.53; median, 0.15). LCVA was correlated with age (r = 0.2634, 95% confidence interval [CI] −0.040–0.522, P = 0.039). Eyes with LCVA better than 0.15 was associated with uncorrected visual acuity (OR, 43.425; 95%CI, 3.729–1250.027; P = 0.010), subjective spherical equivalent (OR, 1.761; 95%CI, 1.255–2.776; P = 0.004), subjective spherical power (OR, 1.616; 95%CI, 1.186–2.433; P = 0.008), and axial length (OR, 0.550; 95%CI, 0.293–0.951; P = 0.032) after adjusted for age and sex. Mean LCVA of less myopic eyes with spherical equivalent > −3.0 diopters was 0.12 ± 0.10 (median, 0.10), and that of more myopic eyes was 0.23 ± 0.14 (median, 0.22) (P < 0.001). Non-pathological myopic eyes exhibited worse LCVA under best correction and had a risk of worse visual ability.

## Introduction

Increase in myopia is a social issue worldwide [1,2]. Preventive therapy for myopia progression in children attracts attention, anticipating reduction of the risk of pathologic myopia which causes substantial visual loss [3–6]. However, whether there is an influence of non-pathologic myopia on visual ability has not been well-documented.

In contrast to pathologic myopia which causes irreversible visual loss due to macular atrophy and neovascularization, non-pathologic myopia may not cause severe visual loss. Most of the non-pathological myopic eyes exhibit normal best-corrected visual acuity (BCVA), 0 in logMAR or better, and evaluated as non-diseased, and healthy eyes in daily clinic.

Low-contrast visual acuity (LCVA) is measured using low-contrast and grey-scale optotypes under best correction in contrast to general BCVA measurement using black optotypes. Because LCVA is measured under more stringent conditions than general BCVA, LCVA is utilized in clinical studies to detect subtle changes in visual acuity [7,8]. LCVA has been used to show the treatment effect of an anti-vascular endothelial growth factor therapy in eyes with age-related macular degeneration, which exhibited relatively good BCVA at baseline compared with the phase III study [7]. In the prospective study, mean general BCVA at baseline was 0.07 in logMAR and good, thus, there had been a concern in detecting the visual improvement after therapy due to ceiling effect. However, visual improvement was clearly shown using LCVA score [7]. It is also used in the studies to show the visual impairment in brain and/or optic nerve damage by classical galactosemia [9] and multiple sclerosis [10,11], as well as in the retinal damage due to central serous chorioretinopathy [12].

In this study, LCVA of healthy eyes with various refraction errors were analyzed to show differences in visual ability. The information will help understand the impact of non-pathologic myopia on vision, and also the usage of LCVA in future clinical studies as well as clinical practices.

## Methods

### Participants

The participants were those who underwent eye medical checkups at Fujita Medical Innovation Center, Tokyo, Japan, from February to December 2025. Those who exhibited BCVA worse than 0 in logMAR were excluded. Those with eye diseases such as glaucoma, age-related macular degeneration, epiretinal membrane, diabetic retinopathy, pathologic myopia defined by presence of myopic maculopathy category 2 or above, or presence of posterior staphyloma [13], macular dystrophy, retinitis pigmentosa, and noticeable cataract, or those with histories of ocular surgeries, such as cataract surgery, implantable contact lens surgery, and laser-assisted in situ keratomileusis, were also excluded. The left eyes of the participants were evaluated.

This study adhered to the principles of the Declaration of Helsinki and was approved by the Fujita Health University Ethics Committee (approval number: HM25–294). Written informed consent was obtained from all the participants to use their data for research purposes.

### Eye examinations

All patients underwent BCVA measurement based on refraction tests, slit-lamp examinations, and fundus examinations. LCVA was tested with the best correction, with the same correction as the BCVA measurement, by showing Landolt C optotypes of Landolt C charts with 6% contrast one by one on the display using SC-1600 (NIDEK Co., LTD, Aichi, Japan). The axial length was measured using ARGOS (Alcon Laboratories, Inc., TX, USA). Cornea/anterior segment optical coherence tomography (OCT) (Tomey Corporation, Aichi, Japan), Heidelberg Spectralis OCT (Heidelberg Engineering, Heidelberg, Germany), and ultrawide field color fundus camera system, Optos (Nikon Corporation, Tokyo, Japan), were used to record images. Subjective spherical equivalent, spherical, and cylindrical powers were evaluated from data obtained by a subjective refraction test performed by the orthoptists after measuring objective refractions by autorefractometer (TRK-2P, Topcon, Tokyo, Japan).

### Statistical analyses

JMP (student edition, JMP Statistical Discovery LLC, NC, USA) was used for all the statistical analyses. Data are presented as the mean ± standard deviation values. Spearman’s rank correlation coefficient analyses, logistic regression analyses, and Mann-Whitney U test were used. Statistical significance was set at P < 0.05.

## Results

Mean age of the 43 participants, all of whom exhibited BCVA better than 0 in logMAR (1.0 in decimal, 20/20 in Snellen chart) was 52.8 ± 6.07 (range, 44–66; [median 52]) years, and 13 (30.2%) were males (Table 1). Mean LCVA was 0.17 ± 0.13 (−0.079 to 0.53 [0.15]) in logMAR; the measurement was performed using Landolt C chart with decimal score, thus the values were originally recorded in decimal score and converted into logMAR. The measured decimal score ranged 0.3 to 1.2, their mean value was 0.70 ± 0.19, and median value was 0.7. Mean spherical equivalent of the eyes was −3.49 ± 2.78 (+1.63 to −10.88 [−3.13]) diopters. All the eyes were phakic with no history of eye surgeries. There were 15 eyes with conus, and 5 eyes with fundas tessellation as observed in fundus photograph although there were no eyes with macular lesions.

**Table 1:** Characteristics of the participants.

age	52.8 ± 6.07 (44–66 [52])
sex (male, %)	13 (30.2)
low-contrast visual acuity in logMAR	0.17 ± 0.13 (−0.079 to 0.53 [0.15])
in decimal	0.70 ± 0.19 (0.3 to 1.2 [0.7])
uncorrected visual acuity in logMAR	0.72 ± 0.48 (−0.30 to 1.30 [0.82])
in decimal	0.36 ± 0.43 (0.05 to 2.0 [0.15])
spherical equivalent (diopter)	−3.49 ± 2.78 (+1.63 to −10.88 [−3.13])
spherical power (diopter)	−3.10 ± 2.78 (+2.25 to −10.25 [−3.00])
cylindrical power (diopter)	−0.78 ± 0.68 (+0.00 to −3.00 [−0.75])
intraocular pressure (mmHg)	15.29 ± 2.00 (13.0 to 20.3 [15.0])
axial length (mm)	25.19 ± 1.26 (22.63 to 27.49 [25.36])
tear breakup time (sec)	5.31 ± 2.80 (2–10 [4.0])
corneal thickness (μm)	535.77 ± 28.33 (458–616 [538]
corneal endothelial cells (cells/mm^2^)	2538.88 ± 306.23 (1501–3116 [2608])
anterior chamber depth (mm)	3.38 ± 0.31 (2.71 to 3.93 [3.42])
lens thickness (mm)	4.30 ± 0.25 (3.84 to 4.98 [4.26])
pupillary diameter (mm)	5.12 ± 0.90 (2.45 to 7.41 [5.14])
central retinal thickness (μm)	263 ± 17.23 (236–306 [260])

Data are expressed as mean ± standard deviation (range [median value]).

**Fig 1:**
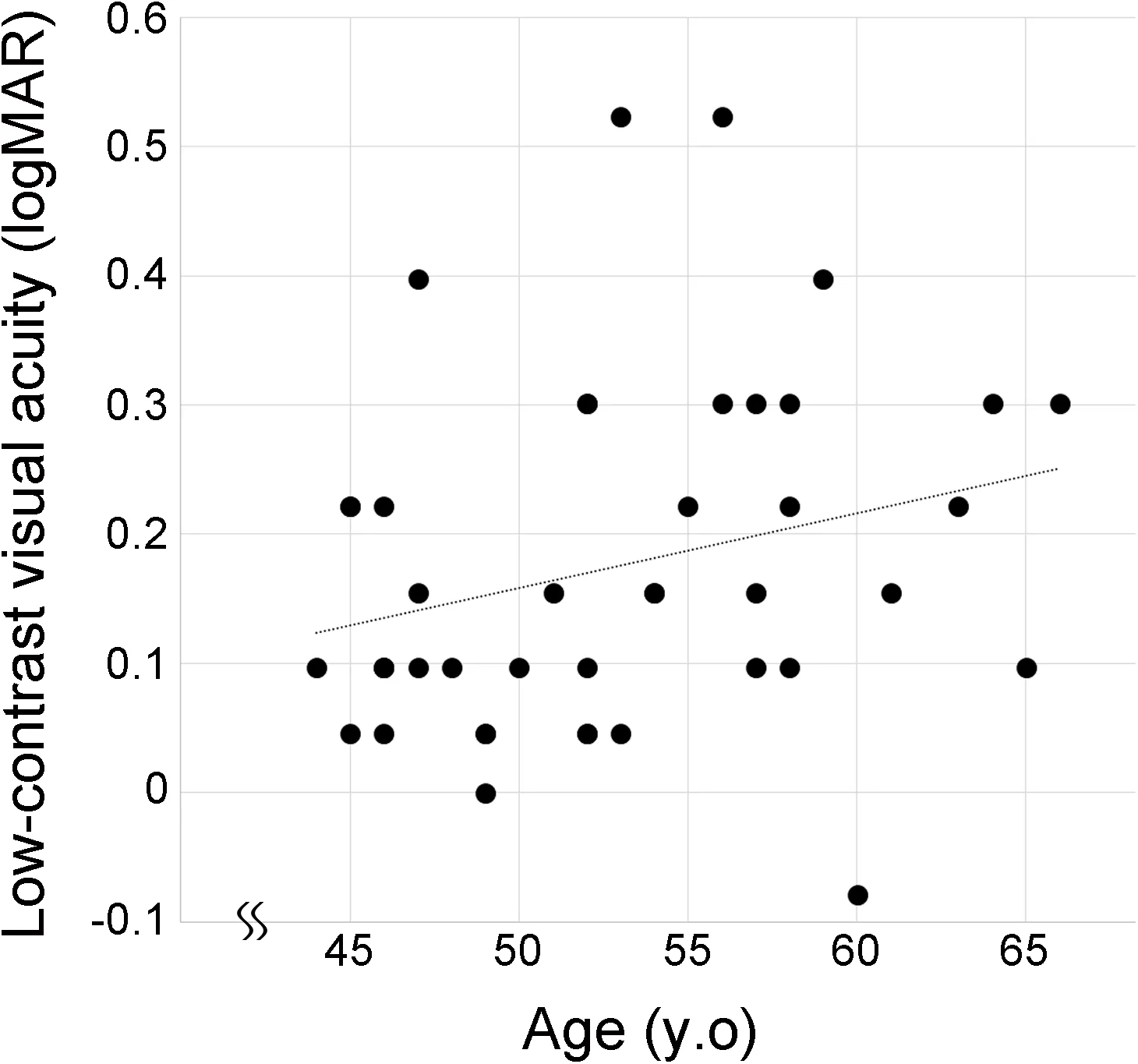
Correlation between low-contrast visual acuity (LCVA) and age. LCVA was positively correlated with age (r = 0.2634, 95% confidence interval [CI] −0.040 to 0.522, P = 0.039).

LCVA was positively correlated with age (Fig 1, Table 2) (r = 0.2634, 95% confidence interval [CI] −0.040 to 0.522, P = 0.039). Other studied parameters, except for lens thickness, were not correlated with age (Table 2).

**Table 2:** Correlations between ocular parameters and age.

	ρ	P
low-contrast visual acuity	0.316	0.039*
uncorrected visual acuity	−0.092	0.555
spherical equivalent	0.048	0.759
spherical power	0.075	0.631
cylindrical power	−0.185	0.236
intraocular pressure	0.029	0.855
axial length	0.108	0.491
tear breakup time	0.194	0.214
corneal thickness (μm)	0.207	0.184
corneal endothelial cells	0.153	0.328
anterior chamber depth	−0.073	0.643
lens thickness	0.503	0.001**
pupillary diameter	−0.294	0.056
central retinal thickness	0.091	0.563

Spearman correlation coefficient. ρ, correlation coefficient. *P < 0.05, **P < 0.01.

Next, the eyes were divided into two groups; eyes which exhibited better LCVA than the median value, 0.15, and those that were worse. The factors associated with better LCVA were analyzed using logistic regression analyses (Table 3). The better LCVA was associated with uncorrected visual acuity (OR, 43.425; 95% CI 3.729–1250.027; P = 0.010), subjective spherical equivalent (OR, 1.761; 95% CI 1.255–2.776; P = 0.004), subjective spherical power (OR, 1.616; 95% CI 1.186–2.433; P = 0.008), and axial length (OR, 0.550; 95% CI 0.293–0.951; P = 0.032) after adjusted for age and sex.

**Table 3:** Factors associated with low-contrast visual acuity in healthy adults.

	Crude					Adjusted with age and sex				
	OR	95%CI			P	OR	95%CI			P
age	0.887	0.783	to	0.988	0.041*	–	–	–	–	–
sex (male, %)	1.600	0.431	to	6.377	0.488	–	–	–	–	–
uncorrected visual acuity	10.589	1.735	to	120.078	0.026*	43.425	3.729	to	1250.027	0.010**
spherical equivalent (diopter)	1.525	1.158	to	2.153	0.007**	1.761	1.255	to	2.776	0.004**
spherical power (diopter)	1.434	1.108	to	1.971	0.013*	1.616	1.186	to	2.433	0.008**
cylindrical power (diopter)	3.358	1.202	to	11.822	0.035*	3.053	1.083	to	11.134	0.056
intraocular pressure (mmHg)	1.136	0.837	to	1.573	0.417	1.143	0.810	to	1.625	0.453
axial length (mm)	0.572	0.317	to	0.956	0.044*	0.550	0.293	to	0.951	0.032*
tear breakup time (sec)	1.059	0.852	to	1.324	0.603	1.150	0.901	to	1.509	0.265
corneal thickness (μm)	1.010	0.988	to	1.034	0.396	1.016	0.991	to	1.045	0.225
corneal endothelial cells (cells/mm^2^)	1.001	0.999	to	1.003	0.046*	1.001	0.999	to	1.004	0.371
anterior chamber depth (mm)	0.611	0.078	to	4.490	0.627	0.246	0.019	to	2.558	0.252
lens thickness (mm)	0.109	0.005	to	1.434	0.118	0.456	0.014	to	10.736	0.635
pupillary diameter (mm)	1.168	0.591	to	2.429	0.655	0.801	0.356	to	1.853	0.592
central retinal thickness (μm)	1.006	0.971	to	1.044	0.723	1.008	0.966	to	1.053	0.719

Univariate analyses and multiple logistic regression analyses after adjusting for age and sex. OR, odds ratio; 95%CI, 95% confidence interval. *P < 0.05, **P < 0.01.

**Fig 2:**
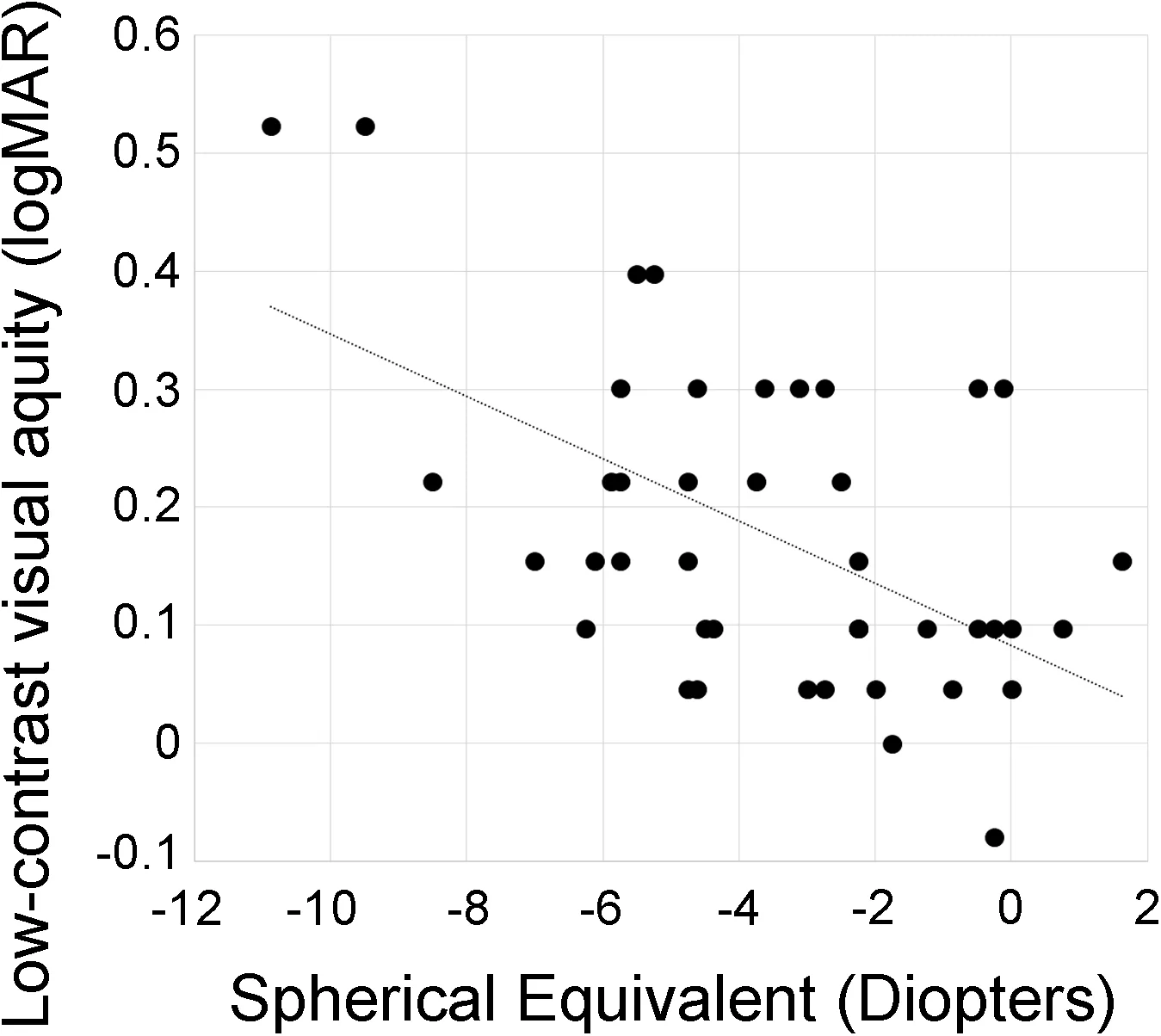
Correlation between low-contrast visual acuity (LCVA) and spherical equivalent (SE). LCVA was positively correlated with SE (r = −0.5501, 95% confidence interval [CI] −0.730 to −0.299, P = 0.0019).

Correlations between LCVA and SE were also significant (Fig 2) (r = −0.5501, 95% CI −0.730– −0.299, P = 0.0019).

We then analyzed the cut-off values of parameters which were related to better LCVA using receiver operating characteristic (ROC) curves. Eyes with better uncorrected visual acuity than 0.70 in logMAR (area under the concentration-time curve [AUC], 0.765), subjective spherical equivalent > −3.0 diopters (0.780), subjective spherical power > −2.5 diopter (0.740), and shorter axial length < 25.3 mm (0.680) more frequently exhibited better LCVA.

The eyes were divided according to the spherical equivalent, and mean values of LCVA in eyes with or without spherical equivalent > −3.0 diopters were analyzed (Table 4). Mean LCVA of the less myopic and hyperopic eyes with subjective spherical equivalent > −3.0 diopters was 0.12 ± 0.10 (median, 0.10) in logMAR (0.79 ± 0.17 [median, 0.8] in decimal), and that of more myopic eyes was 0.23 ± 0.14 (median, 0.22) in logMAR (0.62 ± 0.18 [median, 0.6] in decimal). There was a significant difference between the groups (P < 0.01).

**Table 4:** Mean and median values of low-contrast visual acuity according to the refraction error.

	> −3.0 diopters		≤ −3.0 diopters		P value
Refraction error	mean	median	mean	median	
logMAR	0.12 ± 0.10	0.10	0.23 ± 0.14	0.22	< 0.001**
decimal score	0.79 ± 0.17	0.8	0.62 ± 0.18	0.6	

Data are expressed as mean ± standard deviation and median value. Low-contrast visual acuity was measured using decimal score and converted into logMAR. Mann-Whitney U test. **P < 0.01.

## Discussion

Mean LCVA of healthy adults was 0.17 ± 0.13 in logMAR and median LCVA was 0.15 in logMAR. LCVA was positively correlated with age, and better LCVA was observed in eyes with better uncorrected visual acuity, less myopia, and shorter axial length, after adjusted for age and sex. LCVA was correlated with SE. Less myopic eyes with subjective spherical equivalent > −3.0 diopters more frequently exhibited better LCVA, and their median was 0.10, while that of more myopic eyes was 0.22 in logMAR.

Healthy people are supposed to exhibit a BCVA better than 0 in logMAR; however, LCVA, in which the best correction was provided, but the low contrast optotypes were presented, ranged between −0.079 and 0.53 in logMAR and varied among the healthy individuals. Referring to the median value, an LCVA better than 0.15 in logMAR would be more appropriate to represent eyes with better visual function among healthy adults.

LCVA correlated with age in the current study using 6% low-contrast visual chart. This was consistent with the previous study using 2.5% low contrast chart in which the mean value decreased every decade [14,15]. The current study also showed that the lens thickness increased with age, suggesting a certain influence of lens aging. Given that there were no participants who had already had cataract surgery, age-related progress in lens opacity, if any, could have affected LCVA, although all the eyes exhibited better general BCVA than 0 in logMAR. Alternatively, the number of photoreceptors [16] and retinal pigment epithelial [17] cells, as well as the function of retinal ganglion cells [18] are reportedly reduced as age increases in eyes with no disease, and retinal aging may also have been an effect.

When adjusted for age and sex, eyes with a better LCVA often had less myopia, and this was consistent with the result that eyes with a better LCVA often exhibited shorter axial length. The eyes with better LCVA exhibited good uncorrected visual acuity, most likely related to less involvement of myopia. None of the eyes had any macular lesions; however, the non-pathologic myopia was related to impairment of LCVA even after best correction. In addition, the median values of LCVA in moderate to highly myopic eyes were worse than non-myopic to mildly myopic eyes. Thus, there would be an inferiority in the ability to see in non-pathological myopic eyes. Preventive treatments against myopia progression in children [3–6] may also be valuable for maintaining visual ability under the best correction. Future studies can include LCVA to assess the importance of myopia suppression to preserve better visual ability in non-pathologic myopia.

Myopic eyes exhibit higher-order aberrations (HOAs) that are subtle and complex refractive errors [19]. It is optical imperfections of the eye that alter retinal image ability despite optimal correction of spherical defocus and astigmatism [20]. HOAs can be related to lens shape and position [19]. Among highly myopic eyes < −6.0 diopters in Chinese participants, greater HOAs were associated with reduced visual acuity in simulated night conditions, and decreased contrast sensitivity under mesopic and simulated night conditions [21]. Similar mechanisms may have affected LCVA and visual ability in eyes with spherical equivalent ≤ −3.0 diopters in the current study. Alternatively, central cone photoreceptor density reportedly tends to be reduced as axial length becomes longer [22], which could have influenced LCVA in myopic eyes. Further studies are required to estimate the mechanisms.

Limitations of this study included a relatively small sample size, a retrospective design, and refractive corrections performed with spectacle frames rather than contact lenses. Thus, very highly myopic eyes, such as those with −10.0 diopters, may have been affected by the aberration effects due to thick lenses during LCVA measurement; however, the inferiority of LCVA was also observed in the eyes with −3.0 diopters, and the confounding effect may not be involved in most of the eyes. Evaluation of cataract, if any, was performed only using slit lamp examinations by well-trained ophthalmologists, but not by image data. However, all the participants exhibited no or slight to mild cataract and BCVA of 0 in logMAR or better.

We measured 6% LCVA in healthy participants and found that visual ability varied among individuals without eye diseases. The factors associated with LCVA were not only age. LCVA of better than median value was more frequently observed in eyes with non- to mild myopia (> −3.0 diopters) and shorter axial length (≤ 25.3 mm). Thus, non-pathologic myopia exhibited worse LCVA. The current results may help appropriately evaluate LCVA in clinical practices and clinical trials. Further studies are warranted.

## Supporting information

S1 Data Dataset. (XLSX)
